# Twin Research in the Post-Genomic Era: Dissecting the Pathophysiological Effects of Adversity and the Social Environment

**DOI:** 10.3390/ijms21093142

**Published:** 2020-04-29

**Authors:** Jonathan D. Turner, Conchita D’Ambrosio, Claus Vögele, Martin Diewald

**Affiliations:** 1 Immune Endocrine Epigenetics Research Group, Department of Infection and Immunity, Luxembourg Institute of Health, L-4354 Esch sur Alzette, Luxembourg; 2 Department of Behavioural and Cognitive Sciences, University of Luxembourg, L-4366 Esch-sur-Alzette, Luxembourg; conchita.dambrosio@uni.lu (C.D.); claus.voegele@uni.lu (C.V.); 3 Faculty of Sociology, Bielefeld University, 33501 Bielefeld, Germany; martin.diewald@uni-bielefeld.de

**Keywords:** twins, epigenetics, post-genomics, DNA methylation, early-life environment, epigenetic epidemiology, social adversity, socioeconomic status

## Abstract

The role of twins in research is evolving as we move further into the post-genomic era. With the re-definition of what a gene is, it is becoming clear that biological family members who share a specific genetic variant may well not have a similar risk for future disease. This has somewhat invalidated the prior rationale for twin studies. Case co-twin study designs, however, are slowly emerging as the ideal tool to identify both environmentally induced epigenetic marks and epigenetic disease-associated processes. Here, we propose that twin lives are not as identical as commonly assumed and that the case co-twin study design can be used to investigate the effects of the adult social environment. We present the elements in the (social) environment that are likely to affect the epigenome and measures in which twins may diverge. Using data from the German TwinLife registry, we confirm divergence in both the events that occur and the salience for the individual start as early as age 11. Case co-twin studies allow for the exploitation of these divergences, permitting the investigation of the role of not only the adult social environment, but also the salience of an event or environment for the individual, in determining lifelong health trajectories. In cases like social adversity where it is clearly not possible to perform a randomised-controlled trial, we propose that the case co-twin study design is the most rigorous manner with which to investigate epigenetic mechanisms encoding environmental exposure. The role of the case co-twin design will continue to evolve, as we argue that it will permit causal inference from observational data.

## 1. Introduction

Lifelong health trajectories are influenced by social and societal variables that are subsequently encoded in the epigenome. From the initial aetiology of a negative trajectory, its progression and eventual outcome, social variables exert their influence in a multitude of manners. Unfortunately, the role of the social environment in health trajectories is confounded since the magnitude and relevance of their association varies at different ages and stages of disease progression. This is highlighted by the case of breast cancer. Women with a higher socioeconomic status (SES) have a higher incidence of breast cancer due, in part, to the socioeconomic distribution of risk factors such as an earlier age at menarche, pregnancy later in life, and lower overall fertility. Women with breast cancer and a higher SES, however, have a significantly increased survival rate, in part due to earlier detection, better treatment access, and strong social support [1,2].

The social environment has exaggerated epigenetic and health effects during critical life periods, but is also likely to have a cumulative effect over the life-span. One critical period concerns early life. In developmental health and disease models, the perinatal and early life period is thought to have a predominant impact on the lifelong health trajectory. In part, due to the pioneering work of David Barker, we now have a large set of tools available to investigate the long-term impact of the social environment in early life [3]. Nevertheless, investigating the accumulated effects of the social environment on health or the transmission of the social environment between generations is currently hampered by a lack of toolkits available. There is a need to disentangle sex, age, genetic, family environment and many other un-investigated, and potentially unmeasurable, developmental-experiences from the factors we wish to investigate.

In this article we suggest that with the emergence of new twin research paradigms such as the case co-twin design, studying twins divergent in the stimuli of interest, we can start identifying regions of epigenetic susceptibility and the health consequences of the social environment. We present preliminary evidence that twin lives are not as identical as previously assumed, and that they show sufficient divergence in life experiences so that they can provide an ideal study-pair matched for sex, age, genetics, developmental environment to examine the role of the adult social environment. This in turn will help identify both environmentally induced epigenetic marks and epigenetic disease-associated processes.

## 2. Classical Twin Research

Twin research started in 1875 with the pioneering work of Sir Francis Galton. Despite what would now be considered as terrible bias and poor experimental design, his seminal article “The History of Twins” concluded that “England’s men of genius” were a product of nature rather than nurture. Later, in the 1920s, the classical twin study methodology evolved. Researchers such as Jablonski (1922), Siemens (1924), and Merriman (1924) started taking advantage of the genetic differences between monozygotic (MZ) and dizygotic (DZ) twins to estimate the heritability of traits [4]. These studies established the baseline for twin studies over many decades, relying on the genetic differences between twins to estimate the relative contribution of genetic and environmental influences. Excess similarity for a disease or trait between MZ over DZ twins is classically assumed to depend on genetic makeup rather than environmental exposure since MZ twins develop from a single zygote that splits after development has started, whereas DZ twins share approximately 50% genetic material, as is the case for non-twin siblings as they develop from two independent zygotes. Classical twin studies have proven extremely useful over the past decades, and complementary molecular genetic studies have subsequently identified many disease and phenotype genes. Nevertheless, several key assumptions such as random mating, equal developmental environments, additive genetic mechanisms, and a clear separation of genetic and environmental factors are currently being questioned [5], and the role of twin research is slowly evolving as basic questions such as “what exactly is a gene” are re-visited in the post-genomic era.

## 3. From the Genomic to the Post-Genomic Era

The period since the completion of the Human Genome Project in 2003 to the present day is now referred to as the post-genomic era. This new era has been defined by not only the widespread availability of the fully annotated human genome, but also the genomes of a large number of reference organisms that have changed our view of what a gene is [6].

This change in era reflects the major paradigm shift away from the previous “gene-centred” view (the genomic era) that has occurred [6]. This prior model, in use for the second half of the 20th century, relied on Mendelian segregation of genes to predict disease risk. As we have moved further into the post-genomic era, however, a fundamental re-appraisal of what a gene is has occurred, and long-held dogmas about gene-phenotype interactions have been challenged. The considerable insight gained over this period resulted in the re-conceptualisation of genes away from being a unique functional or molecular entity. This has evolved into something more fluid where segments of DNA function differently depending on physiological or environmental contexts. Post-genomic genes are thought of as units of “genome expression”. In this new definition, the genomic DNA is: an “image of the gene product” that may be sub-divided or spread over a wide genomic area and undergo multiple different regulatory steps, and integrates environmental exposure and epigenetic regulation [6]. Functional genomics has developed as the discipline that analyses and converts genomic data into exploitable information.

As whole-genome sequencing has become readily available, it has now been repeatedly demonstrated that the long-held assumption that MZ twins are genetically identical must be revisited. Repeatedly, fully sequenced the genomes of MZ twins has identified differences in single nucleotide polymorphisms (SNP), as well as structural variants such as copy number variants (CNV), indels and mosaicism. Genomic differences start to appear in the post-split embryo. SNPs are thought to be generated by early mitotoic errors [7]. Structural genomic rearrangements include insertions, deletions (collectively indels), duplications, and inversions. The most common of these are Copy-Number Variants (CNV), where the number of copies a specific, repeated, DNA segments varies. These are under-appreciated since there are thought to be more CNVs than SNPs between unrelated individuals [8]. This is even more true in MZ twins where the number of discordant SNPs is particularly low, but the early post embryonic split allows the early genomic differentiation to occur, and the number of discordant CNVs to rise.

Mosaicism, the presence of two or more genotypes within an individual, is perhaps the major source of genomic differences between twins. Retrotransposons (commonly known as “jumping genes”) are mobile fragments of DNA that can be copied from one genomic location and inserted into another location. This causes mutations and changes the genomic landscape at the new insertion site. Although the large majority of retrotransposons can no longer move, some have retained the ability to move in both the germline as well as in somatic tissues. The work of the laboratory of Faulkner has established that retrotransposons are lifelong mediators of neuronal somatic mosacicism, providing neural diversity during adult neurogenesis, and is necessary to maintain healthy neurogenesis and brain function [9,10]. Female MZ twins have an additional layer of divergence. One of their two X chromosomes is randomly silenced at the 700–1000 cell stage. Although the probability of the maternal or paternal chromosome being inactivated is theoretically equal, the ratio of active maternal or paternal chromosomes will differ between tissues and between twins and is unpredictable [11]. As MZ twins come from a split embryo, the number of cells present when the X chromosome is inactivated is lower, and an increased frequency of skewed X chromosome inactivation has been seen, although this depends on the timing of the embryo split and the relative abundance of cells in the new embryo [12,13].

The importance of the genomic differences must, however, be put into perspective. One recent sequencing study identified an average difference between MZ twins of 14,310 SNPs, 2425 indels, and 16,735 CNVs [14]. These covered a total of 2174 different genes, of which, 37 genes contained all three variant types. However, a total of 335 million SNPs and indels have been identified, and on average an individual has between 4 and 5 million SNPs and short indels [15]. As such, the genetic variation between MZ twins is minimal, although existent, compared to DZ twins or unrelated individuals.

While Mendelian genetics may provide insight into rare single-gene disorders, this is not the case for common complex diseases. With the re-definition of what a gene is, the question has now evolved into “how and under what circumstances is a segment of DNA expressed?” and furthermore “in what ways does the segment function in relation to other molecules and environments, internal and external to the body?”. This has made the prediction of risk problematic for even common complex disorders as even family members sharing a specific genetic variant may well not have a similar risk for future disease, “pulling the rug” from under the long-established twin study rationale. As we move further into the post-genomic era, our understanding of what a gene is and how twins are not genetically identical makes us ask fundamental questions about DNA and inheritance that impact the role of twins in genomic research. This raises the question as to whether Mendelian segregation still hold true. We have long considered genes and their alleles to be what Mendel termed “factors”. As genes have been redefined as fluid entities of genome expression where the different regulatory steps are incorporated it is less dependent on the underlying sequence and has an increased dependence on the environment experienced and epigenetic factors [6]. Previous studies, assuming identical genomes, and not incorporating differences in epigenetic regulation between twins may have overestimated the genetic or heritability component.

## 4. Epigenetics: Moving from Darwinism towards Lamarckism?

The redefinition of what a gene is has been accompanied by the exponential growth in the field of epigenetics. As the field of epigenetics matures, the evidence is growing that Lamarck, and his idea of “acquired traits”, once ridiculed and discredited, may have been (at least partially) correct. Epigenetics describes the control of gene expression through modifications in DNA accessibility, tertiary structure, or through covalent modifications to the DNA itself. Furthermore, these environmentally induced changes are not only reversible and heritable, but leave the underlying nucleotide sequence intact. Epigenetic marks include 5-methyl cytosine patterns on the genomic DNA, post-translational histone protein modifications and their associated changes in DNA structure, and short gene-expression modifying RNA molecules. These epigenetics marks are sensitive to external environmental stimuli. Amongst the many studies demonstrating acquired traits, the most outstanding was the series of reports covering the Dutch Famine in the winter of 1944–1945. These showed the long-term effects of perinatal malnutrition in individuals that would go on to be exposed to overburdening calorie levels. In utero exposure to famine resulted in a lower birth weight. Paradoxically, these individuals went on to develop an obese phenotype [16]. The link was further strengthened when the patterns of postnatal growth were considered. When the growth catch-up during the first years was most rapid, the risk of adult metabolic syndrome and obesity was at its highest [17,18]. On the other hand, foetal over-nutrition has equally adverse consequences. Both pre-pregnancy maternal obesity as well as excessive pregnancy weight gain are associated with increased new-born birth weight [19,20]. As for foetal undernutrition, overnutrition significantly increases the risk of type 2 diabetes and obesity later in life [20]. While it may seem counterintuitive, it would appear that the acquired trait of obesity is linked to a U-shaped birth-weight curve, with both babies born too large or too small are at increased risk of developing obesity [21]. The mechanisms was shown to involve methylation of the IGF2 [22,23], leptin genes [24], and retinoid X receptor-α genes [25]. These studies provide clear evidence that complex disease phenotypes could be programmed in a very short period by factors in the external environment.

## 5. From Genomics to Epigenomics

### 5.1. The Importance of Epigenetics in Human Disease

There are currently two interesting and superficially divergent definitions of epigenetics. Firstly, “the interactions of genes with their environment, which bring the phenotype into being” [26] and secondly the “structural adaptation of chromosomal regions so as to register, signal or perpetuate altered activity states” [27]. As we move further into the postgenomic era, it is becoming clear that both definitions are valid. The first, somewhat simplistic, definition captures the essential interaction with the external environment, whilst the second definition provides the mechanisms through which this gene-environment interaction induces a phenotype and how it may be maintained not only through cell division, but through generations of affected individuals as well. Although it is outside the scope of this review, it should be noted that these epigenetic changes are generally initiated by either the binding of proteins or non-coding RNAs to specific genomic sequences, or the initiation of chromatin remodelling that changes the interaction between proteins, RNA and DNA [28]. These changes result in altered patterns of gene expression independent of the underlying genomic sequence. Once the epigenetic marks are in place, they are effectively a “memory” of the environmental event that is subsequently transmitted through both cell division, and potentially throughout multiple generations of the organism. Furthermore, evidence is now growing that epigenetic modifications may also be reversed by environmental, behavioural or pharmacological interventions [29].

There are life periods in which an organism is particularly sensitive to epigenetic-modifying events that generally coincide with periods of increased cell division and growth such as foetal and early post-natal development as well as the adolescent growth spurt. Overall, these epigenetic modifications encompass the complete diorama of effects from silent, non-functional modifications, to immediate pathogenesis. They may also act over a much longer period of time in which they may lie dormant (or latent) for many years requiring a subsequent environmental insult to crystallise the disease risk initially epigenetically encoded, as originally hypothesised by David Barker as the Developmental Origins of Health and Disease (DOHaD) model. This has enabled us, over the previous decade, to provide molecular evidence on how both nature and nurture are so inextricably linked [30].

### 5.2. Twins as a Resource in Epigenetic/Epigenomic Research

Although we have made considerable headway in understanding complex gene x environment interactions, many challenges remain. One of the key emerging tools concerns the use of twins in epigenetic research. Twin studies have traditionally been used to identify the effects of genetic variants on disease phenotypes, taking advantage of the common early life (in utero and postnatal) environment and fixed genetic similarity (monozygotic twins) or dissimilarity (dizygotic twins). Classical twin studies allow for the deconvolution of the phenotypic variance (or disease discordance) into genetic and environmental components. This has long permitted estimation of both heritability and the genetic contribution to a particular phenotype or disease without necessarily having the genomic sequence. However, there are multiple assumptions in twin-studies that may not be true [31]. With the evolution of rapid and affordable epigenomic technologies the field of epigenomic epidemiology has started to emerge. The ability to dissect genetic and environmental influence in twin studies renders them particularly useful in this growing field, mechanistically linking environmental exposure, gene activity, and disease/phenotype development [32,33]. It should be highlighted that in developing and developed countries non-communicable diseases currently dominate disease patterns. Environmental factors play a primordial role in the pathophysiology of non-communicable complex diseases including cardiovascular disease, type 2 diabetes and depression, and, the power of the co-twin design studies will further help in identifying both environmental risk factors and epigenetic alterations underlying disease pathophysiology [34].

### 5.3. Discordant Twins as a Resource to Reveal Epigenetic/Epigenomic Contributions to Disease: Case Co-Twin Design

There is now a growing body of literature in which disease-discordant twins have been used to identify epigenetic disease associated processes, the so-called case co-twin design [35]. As would be expected, discordant MZ twins allow for the control of age, sex and genetics. Furthermore, the shared in utero and early-life environment can be controlled [35,36]. There is no reason that the case co-twin design cannot be applied to either DZ or MZ twins; however, as MZ twins are genetically identical, any epigenetic and pathophysiological difference must be environmentally induced. Focussing on identical twins then presents a clear epidemiological challenge: finding the environmental exposure that induced the epigenetic changes seen, and that are pathophysiologically linked to disease. In this paradigm, the non-affected MZ twin most likely shared “a common rearing environment during their childhood and adolescent years” [37], providing not only a perfectly matched control, but also permitting control of the many “known–unknown” confounding variables, as well as significantly raising study power.

Becoming familiar with the idea that case co-twin studies can be used to identify environmentally induced disease phenotypes (See Table 1 from [34]) has also turned the tables, and brought about the expectation that twin cohorts will also allow us to see epigenetic differences that are induced by a wide range of environmental influences. Furthermore, with the sensitivity of modern biological techniques we are now able to see genuine biological differences induced by, for example, adverse (psycho)social environments.

Using MZ twins for studying epigenetics has to deal with the fact that on the one hand processes are likely to occur especially in very early life, around birth and early childhood, and again during adolescence, whereas on the other hand discordance in the life experiences of MZ twins is likely to occur later, after having left the parental home. Therefore, studying younger cohorts, who still live together in the parental home or have only recently left it, has the advantage of being closer to most influential experiences rather early in life, whereas this research approach may suffer from not enough discordance in relevant life experiences. The usually low shared environment component in twin-based decomposition of environmental and genetic sources of developmental outcomes and human traits, however, makes evident that there is less uniform impact of environments on individuals than commonly assumed, which is not least due to the fact that a shared environment may impact differently on individuals, even in twins [38]

### 5.4. Discordant Twins (Case Co-Twin Design) as a Tool to for Studying the Social Environment

Case co-twin studies investigating the adult social environment are a recent evolution, with two landmark studies published in 2019. In these studies, inter-twin difference in socioeconomic status were associated with mental health outcomes [39] and differences in the gut microbiome [40].

In studying psychosocial distress, Lam et al. [39] used a divergent twin design, permitting control of unmeasured genetic and environmental confounders and subsequently used “within–between pair regression analysis” that permitted them to examine a potential causal link. This seminal study managed to demonstrate, after controlling for shared environmental and genetic traits, that the higher-SES twin had a lower psychological distress score than their lower-SES twin. Nevertheless, the result was dependent on the measure of SES employed. The association was apparent for the Australian Socioeconomic Index 2006 (AUSEI06), but not for the Index of Relative Socio-economic Disadvantage (IRSD). The association with gut microbiome composition was significantly more robust than for psychological distress. Increased divergence of the microbial composition was associated with higher income levels, area-level SES, and increased educational attainment [40].

These two recent studies mark a ground shift in the twin research paradigm. Together they demonstrate that twin lives are not as identical as thought, and that these divergences in twin lives can be associated with health-related parameters. To further expand on these studies, it is worthwhile examining the sociological and environmental factors that are known to affect the epigenome. This will subsequently allow for the identification of life events in which twins may diverge, and for which there is a strong likelihood that there will be measurable physiological and health-related effects as twins grow apart in adulthood.

### 5.5. Environments and Behaviours Triggering Epigenetic Mechanisms

Twins are born with strongly concordant epigenomes [41]. When DNA methylation levels are discordant, they are at specific loci [42] spread throughout the genome [43] in both MZ and DZ twins. Epigenetic differences present in genetically identical twins at birth imply that the twins experienced differences in the gestational environment [29]. Furthermore, both longitudinal [44] and cross-sectional studies [45,46] have shown that differences increase significantly as twins age. Fraga et al. [45] clearly demonstrated the extent to which the epigenome diverges in monozygotic twins as they age and their lifestyles diverge. Young MZ twins with very similar lifestyles and that have spent their lifetimes together had minimal divergences in their DNA methylation profiles. Older twin pairs, however, that had spent a much lower proportion of their lives together and had considerable lifestyles differences had clear hypo- and hyper-methylation scattered randomly throughout the genome [45]. These age-dependent epigenetic changes most likely play a significant role in healthy aging, reflecting the environment in which the individual lived; however, they are also thought to be involved in aging-associated disorders [47,48]. While there is a growing literature on epigenetic susceptibility to the early life environment, there are many aspects of the environment experienced throughout life that have a significant impact on the epigenome. In the following section these are briefly summarised, with the aim to identify the potential environmental influences that may separate otherwise identical twins.

There is still no commonly applied taxonomy of environmental factors affecting the epigenome but rather several distinctions that are not mutually exclusive to each other but overlap:

- *Physical and social environment*. The physical environment comprises mostly daily stressors for the organism like noise or pollution. The social environment comprises threatening or sheltering and buffering social forces at the levels of individual behaviours, direct social relations, membership in social organizations, and the larger social structure and social institutions of the society.
- *Stressors and resources.* Stressors may stem from the physical or the social environment, e.g., from burdens and aggression in social relationships. Resources can be material (e.g., money) or immaterial (e.g., social recognition), and they may be linked to the individual or family position in the social inequality structure, or they may be located in the social infrastructure at the levels of neighbourhood, region, or welfare states.
- *Daily stressors and singular life course events.* In the social sciences especially transient stressful life events found attention, like the loss of significant others, unemployment, or singular experiences of a violent attack, Other studies found, however, a stronger impact of more enduring daily hassles [49], which got less attention. Moreover, both kinds of experiences are not independent from one another: singular events can alter daily life for quite a long time, e.g., if the loss of a close relationship severely limits the availability of social support thereafter.
- The relative role of *different life course models of risk and adversity,* aiming at identifying mechanisms of cumulative advantage and disadvantage, risk accumulation and risk compensation, the occurrence in sensitive periods, and the duration over lifetime.

Nevertheless, the heterogeneous (biological) epigenetic literature takes many of these elements and tries to break them down into discrete components. This reductionist approach has clearly identified the epigenetic effects of exposure to pollution, smoking, poor nutrition, alcohol, stress, and global socioeconomic status.

*Pollution:* As would be expected, the effects of (air) pollution on the respiratory system are well established although they affect many other organ systems. There is now strong epidemiological evidence linking persistent organic pollutants (POP) to the development of neurological diseases including “neuropathies, cognitive, motor, and sensory impairments; neurodevelopmental disorders such as autism spectrum disorder (ASD) and attention-deficit hyperactivity disorder (ADHD); and neurodegenerative diseases including Alzheimer’s disease, Parkinson’s disease, and amyotrophic lateral sclerosis (ALS)” [50]. Although mechanistic data are scarce and the mechanisms are likely to be pleiotropic and intertwined the available evidence suggests that epigenetic modifications, particularly DNA methylation, are involved [51]. Exposure to particulate matter has been associated with increased methylation levels at the genomic repetitive element LINE-1 [51]. Furthermore, it would appear that pollutant exposure accelerates the epigenetic clock [52], and exposure for as little as 9 days induces significant methylation changes in genes associated with “oxidative stress, cell survival, inflammation, and glucose and lipid metabolism” [53].

*Smoking*: Smoking is a localised form of individual exposure to a series of pollutants. It is the most prevalent (and preventable) cause of mortality and morbidity. As for pollutant exposure, the evidence points to clear epigenetics effects of smoking, particularly on DNA methylation levels (reviewed in [54]). Numerous studies have revealed differential DNA methylation in smokers, in particular at the aryl hydrocarbon receptor repressor (AHHR) locus has been proposed as a proxy biomarker for smoking exposure [55,56]. Mechanistically, changes in DNA methylation were coupled with lower DNA methyltransferases (DNMT) expression in a dose-dependent manner after tobacco smoke exposure [57]. Furthermore, epigenetic mechanisms including, histone deacetylase 2, and histone acetyltransferase levels were rapidly altered after exposure to tobacco smoke [58,59]. The children of smoking mothers have been shown to have accelerated epigenetic clock at birth and this difference rose during childhood and adolescence [60].

*Nutrition*: As highlighted in the introduction, nutrition has an important impact not only on the epigenome, but also on the overall phenotype. In addition to the epidemiological evidence linking nutrition to long-term phenotype development, there are specific nutrients that have well established effects on both epigenetic mechanisms as well as specific epigenetic markers. For example, folate, a water-soluble B vitamin, is necessary for the synthesis of AdoMet in the DNA methylation pathway [61,62] involved in the synthesis of AdoMet and dietary folic acid supplementation results in increased DNA methylation levels at multiple genes involved in both the developmental and ageing processes [63]. Many other methyl donor nutrients such as choline can also alter the DNA methylation status and subsequently impact gene expression [62]. The availability of methyl donors from nutrients throughout pregnancy is primordial for foetal development. Reduced methyl donor levels have lifelong consequences for the offspring’s health, disease susceptibility and cancer risk. In one pre-clinical study, maternal diet with limited methyl donors around the period of conception affected not only the offspring’s DNA methylation patterns but also altered the phenotype [62] (Reviewed in [64]).

*Alcohol consumption:* Epigenetic effects of ethanol occur through an exquisite mix of DNA methylation and histone acetylation [65]. Acute ethanol exposure activates histone acetyltransferases (HATs) while concurrently inhibiting histone deacetylases (HDACs) [65]. These enzymes leave active epigenetic marks that induce chromatin remodelling and change gene expression [65,66]. More recently, Mews et al. (2019) showed that acetate from hepatic ethanol metabolism directly binds to neuronal chromatin [67]. Here, it is converted to acetyl-CoA by acetyl-CoA synthetase 2(ACSS2), and then serves as a direct substrate in histone acetylation. Furthermore, DNA methylation changes have been observed in brain, buccal tissue and blood [68,69,70,71]. DNA methylation and histone modification profiles were different but consistent. Decreased methylation levels occurred concurrently with H3K4me1 and H3K79me2, both histone modifications that are characteristic of enhancer or activator regions. Inversely, increased methylation levels, observed within gene bodies, coincided with regions enrichment in H3K36me3, both modifications regularly observed in actively transcribed genes [70]. Maternal alcohol consumption during pregnancy has been shown to be associated with decelerated epigenetic clock at birth and this difference disappeared during childhood [60].

*Stress:* Exposure to stress in early life is a well-established paradigm leading to an increased lifelong risk of negative health outcomes, especially in mental disorders and metabolic diseases. The epigenetic effects of early-life stress have been well-documented and reviewed elsewhere [72]. In the context of twin research, where the early life environment is shared, but the environment diverges later, stress exposure in adulthood is more relevant. In a similar manner to early-life, adult psychosocial stress is a major disease risk factor that is epigenetically encoded [73]. There are now several studies that demonstrate an epidemiological link between psychosocial stress exposure and DNA methylation patterns. Only considering the Post-Traumatic Stress Disorder (PTSD) literature (reviewed in [74]), the HPA-axis is clearly epigenetically dis-regulated, with increased HFBP5 methylation levels in survivors of the Holocaust [75] and altered GR promoter methylation [76,77]. PTSD is accompanied by low-grade inflammation that is associated with methylation of inflammatory genes (glucocorticoid receptor, TLR2, ICAM-1, inducible NOS, interferon-γ, and interleukin-6) [78,79], and long-term changes in circulating pro-inflammatory cell numbers [80,81,82].

*Socioeconomic Status:* Socioeconomic status (SES) is a blunt but all-encompassing measure of an individual’s environment [83]. Not only does it cover an increased burden of financial and psychosocial stressors, it also encompasses many of the environmental factors such as irritants, pollutants, and lifestyle that have been considered above (e.g., alcohol, smoking, nutrition, and BMI). Many of these factors are individually encoded epigenetically, and this is reflected in the epigenetic imprint of both early-life and late-life SES. It is commonly assumed that the effects in early-life are more significant than during adulthood, although this remains to be shown. Indeed, global methylation levels were reduced in low SES adults and were strongly associated with markers of inflammation such as fibrinogen and IL-6 [84]. Furthermore, twin studies have shown that differences in the epigenome increase with age suggesting that SES throughout life influences the epigenome [44,85,86]. There is also growing evidence that the immune system is epigenetically sensitive to SES. Exposure to low SES changes the fundamental transcriptional profile of T-helper cells. This represents the fundamental identity of immune cells, and we have suggested that the immune system not only protects the individual from pathogens, rather, it is involved in an individual’s adaptation to its immediate environment [83,87]. The role of the immune system is further reinforced by the increased cortisol levels and pro-inflammatory profile seen in low SES together with the methylation of genes involved in inflammation such as NFATC1 NLRP12, CCL1, CD1D, TLR3 [88,89].

## 6. Position and Hypothesis

We hypothesise that twin lives are not as identical as generally thought. Furthermore, we hypothesise that divergence in events that occurred and the meaning attached to the event by the individual (salience), starts as early as during adolescence, and further diverging as they leave the parental home. Furthermore, we hypothesise that discordance will be found not only for individual events, but also the salience of shared events may differ, even among MZ twins. These divergences will cover a range of environmental factors that may act, at the epigenetic level, to induce long-term phenotypic differences. We propose that if twins do diverge over their life-course to a large-enough extent, the evolution of divergent co-twin study designs will allow us to exploit these divergences. This will permit us to investigate the role of the adult social environment and its role, in particular when there is clear social adversity, in determining lifelong health trajectories.

## 7. From Theory to Practice

### 7.1. Proposed Operational Definition of Adversity

Linking epigenetic processes to the life course and individual development requires an understanding of the individual being inseparably (which is the original notion of term “individual” derived from Latin) shaped by intertwined biological, psychological, and social processes, be it that biological mechanisms influence individual development and life courses, be it that social experiences and individual behaviours trigger biological mechanisms. Both directions of influence have to be considered when defining and operationalizing adversity.

Following our review of the environmental events that may affect the epigenome (pollution, smoking, nutrition, alcohol, stress and SES), we propose nine identifiable and quantifiable adverse life events, all intimately linked with these exposures, that are commonly measured in social science cohorts. These advents should be applicable to only one member of a twin pair that are associated with these known epigenetic factors. Based on prior literature [90,91,92,93,94] we propose the following social experiences:

(i) Serious negative health events such as illness or disability;
(ii) Been made redundant, becoming unemployed;
(iii) Being demoted or having an imposed reduction in working hours;
(iv) Serious relationship problems or separation from partner;
(v) Been the victim of a serious crime;
(vi) Being the victim of interpersonal psychological or physical violence, as in bullying or intimate partner violence;
(vii) Experiencing poverty, having a major financial problem;
(viii) Failing a formal education/training program;
(ix) Returning to the parental home/having a child return to your home.

Primarily, these experiences refer to focal individuals as experienced by themselves. However, especially for children and adolescents some of these experiences could also matter if experienced by very close relations, first of all parents or other persons in the family. Thus, also the experience of poverty or unemployment by parents, or parental separation are part of this compilation.

These measures should be quantified in addition to more enduring health-threatening behaviours, like smoking or drinking, and chronic stress levels and diseases. These indicators of life circumstances that may trigger epigenetic reactions should be complemented by indicators or resources that are helpful to compensate adversity and thus may prevent epigenetic reactions. Socioeconomic status has repeatedly been shown to moderate epigenetic reactions to stressors and trauma. Additionally, social relations and social participations that may compensate failure in life domain by social support and experiences of worthiness and success in other life domains.

### 7.2. Measuring Socioeconomically Divergent Twins

To assess to which degree there is divergence in MZ twins already in adolescence and post-adolescence, we examined data from the German TwinLife study [95,96,97,98,99] for the cohorts at the ages of 11, 17, and 23–24 during the time of the first interview (2014/2015). The youngest cohort, at the age of 5 during the first interview, was omitted because for them mostly a parent provided the information about their life experiences. TwinLife is an ongoing long-term behavioural genetics cohort conceived to investigate the effects of social inequality in over 4000 twin families. Data available include a broad range of longitudinal environmental measures that are uniquely relevant for both epigenetic and gene expression studies [96,97,99]. One of the major findings from TwinLife has been the confirmation of the Scarr-Rowe-interaction. The results show that parental social disadvantage (low SES) compromises the extent to which a child’s genetic potential for both educational attainment [95] and IQ [97] are realized, whereas socially advantaged parental homes (high SES) enhance it. TwinLife is, worldwide, among the genetically informed studies with the most comprehensive social science information permitting examination of gene-environment-interactions as well as data about critical life events and their perception, not as clinical but self-report health indicators, behavioural problems, deviant behaviours and a wide range of unequal living conditions and life course outcomes [98].

These data provide us with the possibility to compare divergence for everyday living conditions and single events for the age range of 11 to 24 years. Moreover, we additionally investigated the individual perception of the events assumed to be stressful. Thus, even if the twins shared the same event they could differ in their individual perception of the same event or the same living conditions. We do this only for twin pairs for which we have valid information from both twins. In the two younger cohorts the twins still almost completely lived in the parental household, thus sharing the same environment to a great extent. The oldest twins (cohort 4) are observed for the first time prior to or shortly after leaving the parental home and pursuing tertiary education or establishing themselves in the labour market. Because some of these experiences apply to quite rare events, we display results as number of cases and not percentages, in order to demonstrate actual possibilities to include them into multivariate analyses. Occasionally, we refer to percentages in the text. We calculated these results also for DZ twins but do not display results, since the focus is here on the use of MZ twins for epigenetic analyses. On average, discordance among DZ twins was between 1.2 to 2 times higher than for MZ twins. Table 1 reports the experience of several events and the experience of discrimination. We also asked for the criteria of discrimination. However, numbers are far too low to be exploited for analyses on discordance. Table 2 reports the individual evaluation of the same experiences (with the exception of dropping out from vocational training or university being not part of this item battery) as positive or negative, ranging from “very negative” (minus 3) to “very positive” (plus 3) with 0 as neither positive nor negative. In addition, everyday experiences are included in the form of individual perceptions of the same chaotic home environment. Finally, Table 3 displays self-reports of a number of physical and mental health impairments and illness.

With respect to the divergence of the everyday experience of discrimination and the life events occurring that may cause strains for respondents, Table 1 shows that already for the two cohorts where twins still live in the parental household there is a considerable degree of discordance, though in general discordance is highest in the oldest cohort. For single events, however, as well as for summarizing discordance across all events under consideration, there is no threshold between those still living in the parental household and the oldest cohort where only few twins still live with their parents. Moreover, also for uncommon events the number of cases seems to allow for discordant twins being used for progressive analyses. It is no surprise that for individual experiences discordance is much higher than for events in the proximal social environment. Put differently, for events concerning parents or other family members, one may ask whether there should be divergence at all. We interpret divergence in reporting parental or other family members’ experiences as stemming from interindividual differences in the salience of these events or a certain latitude in defining who belongs to the family. However, without additional information this question has to be left open.

Table 2 compares the discordance in the salience of these events across MZ twins. The numbers are considerably smaller than for the events themselves, which could suggest using this information in a rather cumulative than specific way. Again, there is no evidence that living in the same household leads to only negligible discordance compared to those living in different households. Moreover, the perception of the same parental household differs between MZ twins to a non-negligible degree as well: for all three cohorts, discordance is approximately 16 per cent across all single items together.

Finally, despite the same genetic makeup for MZ twins, there is also discordance for a number of physical and mental health impairments (see Table 3). Numbers are clearly too small for health impairments like cancer or stroke that are, as expected, rare events in such a young cohort. However, though concordance largely prevails overall, especially for asthma or migraine a considerable number of discordant twins, and also for chronic back problems and depressive symptoms, a considerable number of MZ twin pairs is discordant. Summing up all single health indicators, for approximately one third of MZ twins one of the twins reports at least one health impairment, and the other one does not.

## 8. Conclusions

In this paper we develop the hypothesis that the case co-twin study paradigm may be an elegant manner in which to evaluate the epigenetic and long-term health effects of the adult social environment. Data from the TwinLife cohort clearly supports our initial hypothesis that twin lives are more divergent than previously assumed. Twins as young as 11 years of age were discordant for a number of physical and mental health impairments. In approximately one third of MZ twin pairs one of the twins reports at least one health impairment, and the other one does not. Measuring twins experience of life events that may cause strain revealed that a considerable degree of discordance can be found, starting when twins are pre-adolescent and increasing as they age. It would appear that the number of cases we identified will, in the future, allow us to use twin cohorts to follow this increasing divergence in longitudinal or progressive analyses. While twins were younger, they did not share the same perception of their shared environment, and similarly, they diverged in their reporting of parental or other family members’ experiences. This stems most probably from interindividual differences in either the salience or interpretation of these events, or a certain latitude in defining who belongs to the family. Discordance in the salience of specific images or situations has previously been reported in twins. Indeed, fMRI studies have demonstrated differences in the response to visual food cues both before and after eating in MZ twins divergent for restrained eating. In this paradigm, the divergent intent to exert cognitive control over food intake in twins induced divergent fMRI images in response to visual food cues [100]. This suggests that simple visual clues enhance the salience for susceptible individuals, and were able to differentially activate brain regions associated with emotional arousal and increased attention in phenotypically divergent MZ twins.

Our starting point for this hypothesis was that in the post-genomic era genes are being re-defined and somewhat undermining classical twin studies since the underlying sequence is becoming less important. This move to the post-genomic era has also provided a significant confounder–that MZ twin genomes are not as identical as previously thought. The data available suggests that the differences are small, but reliably detectable. Furthermore, as our epigenetic knowledge increases, it is become clear that this plays a further compounding role as genetic inheritance in twins may differ due to epigenetic factors. However, the difference between MZ twin genomes and between unrelated individuals are many orders of magnitude lower.

As hypothesised, we conclude that twin lives are not as identical as generally thought. Data from the German TwinLife registry also confirms that divergence in both the events that occur and the salience of the events for the individual starts as early as during adolescence, visible in twins aged 11 years. The divergences we observed do indeed cover a range of environmental factors that may act, at the epigenetic level, to induce long-term phenotypic differences. The phenotypic differences were observed for a number of physical and mental health impairments. There is now compelling evidence that case co-twin study designs are a powerful tool to detect and evaluate subtle environmental effects. Recruiting divergent twins will allow us to exploit these divergences, permitting the investigation of the role of the adult social environment, in particular when there is clear social adversity, in determining lifelong health trajectories. It may be possible to extend this further, and in cases where the same traumatic event was experienced, investigate the epigenetic and health consequences of the individual salience, crystallising the psychological impact of an event. In cases like social adversity where it is clearly not possible to perform a randomized-controlled trial, we would argue that the case co-twin study is the most rigorous manner in which to investigate epigenetic mechanisms encoding environmental exposure. They provide an ideal study-pair matched for sex, age, genetics, family environment as well as countless unmeasured developmental-experiences. Furthermore, the role of the case co-twin design will continue to evolve, as we think that it will permit causal inference from observational data.

## Figures and Tables

**Table 1:** Discordance between monozygotic (MZ) twins: occurrence of life events (based on valid information for both twins only) (N).

	Age 11		Age 17		Age 23–24	
Life Events ^1^	Discordant	Concordant	Discordant	Concordant	Discordant	Concordant
Experience with discrimination			122	844	130	906
Own separation	NE	NE	200	368	162	244
Separation parents	10	514	34	534	16	390
New relationship parents	42	482	34	534	32	374
Own money worries	NE	NE	38	530	80	326
Money worries family members	28	466	88	470	96	308
Own accident/illness	184	340	164	404	116	290
Accident/illness family members	146	370	190	380	124	282
Own job loss	NE	NE	28	540	48	356
Job loss parents	50	470	90	478	72	334
Dropping out from voc. training/university			12	108	76	230
Victim of violence	34	488	66	504	72	334
Victim violence family member	46	464	100	468	98	306
Death family member	82	438	104	464	72	334
Discordance in number of events experienced ^2^	88	198	184	254	162	192

^1^ The question was whether respondents experienced these events during the past 12 months or before. ^2^ No deviation or only 1 event coded as concordant, more than 1 event as discordant. NE–Not evaluated.

**Table 2:** Salience of living conditions and evaluation of life events (based on valid information for both twins only) (N).

	Age 11		Age 17		Age 23–24	
	Discordant	Concordant	Discordant	Concordant	Discordant	Concordant
**Evaluation of life events ^1^:**						
Own separation	NE	NE	54	54	68	74
Separation parents	10	26	34	86	24	74
New relationship parents	6	24	30	88	36	60
Own money worries	NE	NE	6	4	8	22
Money worries family members	8	14	10	28	12	40
Own accident/illness	18	44	16	28	8	24
Accident/illness family members	24	118	38	174	24	170
Own job loss	NE	NE	2	0	6	6
Job loss parents	14	22	18	30	24	40
Victim of violence	0	4	6	14	8	14
Victim violence family member	4	12	4	20	8	14
Death family member	40	214	36	312	46	222
**Chaotic home (CHAOS scale)** ^2^						
Regular bedtime routine	232	556	NE	NE	NE	NE
Cannot clearly think at home	180	580	176	788	372	770
Chaotic home	138	648	172	806	398	630
Everything under control	128	652	96	870	402	622
TV almost always on	174	630	182	800	422	616
Quiet atmosphere	228	564	164	810	474	556
CHAOS discordance scale^3^	110	582	150	794	162	842

^1^ Respondents were asked to evaluate these events from -3 = very negative over 0 = neither negative nor positive to 3 = very positive. More than 1 point difference is counted as discordant. Only those were asked who previously said that they experienced the event (for the oldest cohort asked retrospectively). ^2^ Respondents were asked to evaluate these items as 1 = completely false, 2 = largely false, 3 = partly true, 4 = largely true, or 5 = completely true. ^3^ Scale comprises 30 points for the youngest cohort and 25 for the two older ones. More than 7 or 9 points difference, respectively, are counted as discordant. NE–Not evaluated.

**Table 3:** Self-report of physical and mental illness (based on valid information for both twins only) (N).

	Age 11		Age 17		Age 23–24	
Self-Report Illness (Reported/Not Reported)	Discordant	Concordant	Discordant	Concordant	Discordant	Concordant
Sleeping disorder	42	952	48	996	42	952
Diabetes	4	990	4	1040	4	990
Asthma	100	894	100	944	100	894
Heart disease	36	958	26	1018	36	958
Cancer	8	986	14	1030	8	986
Stroke	6	988	2	1042	6	988
Migraine	54	940	92	952	54	940
High blood pressure	22	972	34	1010	22	972
Anxiety disorder	28	966	32	1012	28	966
Alcohol addiction	6	988	0	1044	6	988
Depression	36	958	76	968	36	958
Degenerative joint disease	26	968	38	1006	26	968
Chronic back problems	58	936	56	988	58	936
Physical disability	22	972	24	1020	22	972
Other physical/mental illness	114	880	170	874	114	880
No illness/disease diagnosed	326	668	358	686	326	668
